# Worry related to climate change in Brazilian adults

**DOI:** 10.47626/2237-6089-2024-0905

**Published:** 2025-09-18

**Authors:** Maria Nieves, Karen Jansen

**Affiliations:** 1 Centro de Ciências da Saúde Universidade Católica de Pelotas Pelotas RS Brazil Centro de Ciências da Saúde, Universidade Católica de Pelotas, Pelotas, RS, Brazil.

**Keywords:** Climate anxiety, eco-anxiety, climate change, climate crisis, adult

## Abstract

**Objective:**

Climate change is happening and feeling anxiety can be seen as a natural response to it. Climate anxiety is worry about the climate crisis and can be related to specific emotions and thoughts. The aims of this study were to assess the prevalence of worry about climate change and describe the emotions and thoughts associated with it in Brazilian adults.

**Methods:**

A cross-sectional study was conducted with participants aged 18-42 years (n = 323). Data were collected online using a questionnaire developed by Hickman et al., which assesses worry, thoughts, and feelings about climate change.

**Results:**

88.5% of the participants were worried about climate change. Those who were worried reported feelings of sadness, powerlessness, fear, and anxiety more often. The most frequent thoughts were “People have failed to take care of the planet,” “The future is frightening,” “My family’s security will be threatened,” and “Humanity is doomed.”

**Conclusion:**

In this sample, the majority of the individuals were concerned about climate change and showed more negative emotions and thoughts when compared to individuals who were not concerned. Future studies should take care not to interpret natural worries and anxiety responses to climate change as pathological.

## Introduction

There is scientific consensus that global warming is happening.^1^ Anyone with access to information about climate change is potentially susceptible to experiencing an anxiety response.^2^ Such reactions should not be immediately seen as pathological, as they can serve as motivation to find solutions to mitigate climate change.^2^ However, when anxiety is dysregulated, it can lead to psychological suffering and maladaptive behaviors.

Climate anxiety is an emerging construct, and has been understood as distress and worry related to climate change. It can be connected to emotions such as fear, anger, grief, despair, guilt, and shame.^3^ Substantial levels of distress related to climate change have been reported globally.^2-5^

Since only one study was found that included Brazilians as part of its sample,^3^ this study aimed to fill the gap in the literature (both local and international) by assessing the prevalence of worry about climate change and describing the emotions and thoughts related to it in Brazilian adults.

## Methods

A cross-sectional study was conducted with Brazilian participants aged 18-42 years and living in Brazil. Data were collected online, through self-report, from July to August of 2022.

The questionnaire used was developed by Hickman et al.^3^ and assesses worry, thoughts, and feelings about climate change using five questions. The first question is answered using a Likert scale ranging from “not worried” to “extremely worried.” The remaining questions are answered with the response options yes/no/prefer not to say. Answers to the first question were dichotomized as no (not worried/a little) or yes (moderately/very/extremely worried).

Exposure variables were obtained using a questionnaire assessing gender, race/ethnicity, age, education, region of residence in Brazil, income, source of information about climate change (social media, TV and radio, scientific articles), frequency of receiving climate change information (very often, often, sometimes, rarely), and perceived knowledge about the climate crisis, assessed using a questionnaire created by the authors. This questionnaire comprises 10 questions assessing how much respondents thought they knew about climate change and its causes and consequences. Questions are answered using a four-point Likert scale ranging from “I’m sure” to “I’m not sure.” The questions are as follows: 1) I think human action is the main cause of the climate crisis; 2) I know what the greenhouse effect is; 3) I know why the glaciers are melting; 4) I know why it is important that there is less plastic production; 5) I think there is a link between deforestation and the climate crisis; 6) I know why sea level rise poses a risk to continents/islands; 7) I realize that every year it gets warmer and I understand the connection with the climate crisis; 8) I think waste production influences the climate crisis; 9) I know there is a link between pollution and global warming; 10) I think big polluting companies are partly responsible for the climate crisis.

Statistical analysis was performed using SPSS 26 software. Variables were expressed as absolute and relative frequencies. Analytical hypotheses were tested with the chi-square and *t* tests, considering p < 0.05 as significant.

### Ethical considerations

The Research Ethics Committee at the Universidade Católica de Pelotas approved the study, under protocol number 5.412.278. All participants included in the study provided written consent.

## Results

In total, 323 subjects were eligible to participate in the study. Most of the sample were women (68.7%), 18 to 26 years old (60.5%), from the South of Brazil (75.2%), were undergraduates (47.1%), had income ranging from one to five times the minimum wage (81.7%), and self-declared as white (83.2%).

In this sample, 88.5% were worried about climate change (69% very worried or extremely worried; 19.5% moderately worried), and 11.5% were not worried (or a little worried).

Worry was more prevalent among subjects who received or read news related to climate change more frequently (p = 0.001), with no statistically significant difference between the sources of such news. Moreover, people who were worried about climate change declared greater knowledge about the climate crisis (27.25±3.03) when compared to people who were not worried (23.92±5.13; p < 0.001).

None of the sociodemographic variables were statistically associated with a higher prevalence of worry about climate change.

Feelings of sadness (85%), powerlessness (79.7%), fear (78.3%), and anxiety (67.4%) were reported more often among those worried about climate change. Comparing the two groups, all the feelings assessed were reported more often among those who were worried with climate change, except indifference and optimism, which were reported by 17.6 and 16.7% of those not worried and 7.2 and 4% of worried participants, respectively. All the feelings assessed were statistically different between the two groups, with the exception of grief (p = 0.073) (Table 1).

**Table 1:** Frequency of thoughts and feelings/emotions reported by people who are worried about climate change (n= 323)

	Frequency among people worried about climate change n (%)	Frequency among people not worried about climate change n (%)	p-value
Thoughts			
I’m hesitant to have children	152 (55.9)	10 (27.8)	0.002*
Humanity is doomed	207 (76.7)	17 (45.9)	0.001*
The future is frightening	256 (91.8)	24 (66.7)	0.001*
I won’t have access to the same opportunities my parents had	162 (58.5)	16 (43.2)	0.079
My family’s security will be threatened (economic, social, physical)	218 (78.1)	20 (54.1)	0.001*
The things I most value will be destroyed	186 (68.4)	16 (44.4)	0.004*
People have failed to take care of the planet	271 (97.5)	29 (80.6)	0.001*
			
Feelings/emotions			
Sadness	238 (85.8)	16 (43.2)	0.001*
Powerlessness	224 (79.7)	17 (47.2)	0.001*
Fear	220 (78.3)	15 (40.5)	0.001*
Anxiety	190 (67.4)	11 (29.7)	0.001*
Anger	177 (63.0)	8 (21.6)	0.001*
Helplessness	157 (56.5)	9 (24.3)	0.001*
Guilt	150 (54.2)	6 (16.2)	0.001*
Shame	127 (45.5)	10 (27.0)	0.033*
Depression	91 (33.3)	3 (8.3)	0.002*
Despair	89 (32.1)	2 (5.4)	0.001*
Grief	68 (24.5)	4 (11.1)	0.073
Hurt	52 (19.0)	1 (2.7)	0.013*
Indifference	20 (7.2)	6 (17.6)	0.037*
Optimism	11 (4.0)	6 (16.7)	0.002*
			
Total	286 (100)	37 (100)	

“Prefer not to say” responses are excluded from the analysis.

* Statistically significant.

The most frequent thoughts among the people who were worried about climate change were “People have failed to take care of the planet” (97.5%), “The future is frightening” (91.8%), “My family’s security will be threatened (economic, social, physical)” (78.1%), and “Humanity is doomed” (76.7%). All the thoughts assessed were statistically different between the two groups, with the exception of “I won’t have access to the same opportunities my parents had” (p = 0.079) (Table 1).

Finally, 34.6% of the people who reported climate change worry said they believed that their feelings about climate change negatively affect their daily life.

## Discussion

In this study, 88.5% of the sample reported worry about climate change, which is in line with previous findings reporting worry in more than 50% of sample participants.^3,6^

People who were worried reported a higher frequency of access to climate crisis-related news, as has already been suggested,^4^ although in this study there was no statistically significant difference in terms of information sources, unlike previous findings suggesting that media coverage of climate change directly affects the level of public concern.^7^

Those who were worried also reported greater perceived knowledge related to climate change, when compared to people who were not worried about the climate crisis, in line with findings from other studies.^8,9^ However, it should be noted that both the studies mentioned were about educational level, whereas this study analyzed participants’ perceptions of their knowledge regarding the climate crisis, and not their academic level.

All feelings and thoughts except grief and “I won’t have access to the same opportunities my parents had” were statistically different between worried and not worried participants, meaning that, in this sample, there was an association between being worried and having those feelings and thoughts.

Feelings of sadness, powerlessness, fear, and anxiety were reported more frequently by those who were worried about climate change, which differs a little from previous results, where the more frequently reported feelings were fear, sadness, anxiety, and anger.^3^

The most frequent thoughts were “People have failed to take care of the planet,” “The future is frightening,” “My family’s security will be threatened,” and “Humanity is doomed.” The first and second most frequent thoughts found are the same as previous findings.^3^ However, there are differences: here, the thought about family security appears in third place whereas in the prior study it was not one of the most frequently endorsed thoughts^3^; and the idea of not having “access to the same opportunities that my parents had” was not statistically different between groups in the present study’s results, showing that there is possibly no association between this kind of thought and worry about climate change, i.e. it occurs irrespective of the climate crisis. In the other study, with a North-American and European sample, this thought was the fourth most frequently endorsed.^3^ This raises the question of cultural differences between developed and developing countries and their relationships to climate change, worry, access to opportunities, and thoughts about the future.

None of the sociodemographic variables were statistically associated with a higher prevalence of worry about climate change. In one prior study, older people were more likely to have skeptical views, perceived climate change impacts as less negative, and had lower levels of worry about climate change than younger respondents.^8^

The finding of negative impact on daily life in 34.6% of the sample was lower than some results reported previously^3^ and higher than others.^10^

The results of this study in Brazil indicate that there is worry about climate change, as well as thoughts and emotions associated with it. However, the findings should be interpreted with caution, since this study has limitations. Non-probabilistic sampling and use of a non-standardized and not previously validated assessment instrument may have generated an overestimation of the outcome. The survey title may also have biased responses, since there is a possibility that only people already concerned about the climate crisis were interested in participating in the survey when they found it online. However, given the absence of Brazilian studies on the subject, the data presented here serve as a preliminary finding and, notwithstanding the limitations and possible cultural differences, fill a gap in the literature and contribute to the overall understanding of the impacts of climate change on the mental health of populations.

Since studies on climate anxiety are still scarce and what is pathological or not is not yet well defined,^11^ more studies are needed on the subject to delineate the distinction between natural worries and anxiety responses in the face of climate change and a perhaps pathological and paralyzing response. This distinction is also important to enable creation of interventions to address climate change anxiety.

It is also worth mentioning that since the climate crisis is a global issue, it is urgent that governments take real actions to mitigate and prevent the impacts of climate change, not only on people’s mental health, but also on the environment as a whole.
